# Gender-Specific and U-Shaped Relationship Between Serum Uric Acid and All-Cause Mortality Among Chinese Older Adults: A National Population-Based Longitudinal Study

**DOI:** 10.3389/ijph.2023.1605934

**Published:** 2023-05-03

**Authors:** Yinqiao Dong, Zhiqiang Wang, Suping Wang, Ruijie Chang, Yujie Liu, Rongxi Wang, Hui Chen, Shangbin Liu, Changlin Qian, Yong Cai, Fan Hu

**Affiliations:** ^1^ School of Public Health, Shanghai Jiao Tong University School of Medicine, Shanghai, China; ^2^ Hongqiao International Institute of Medicine, Tongren Hospital, Shanghai Jiao Tong University School of Medicine, Shanghai, China; ^3^ Center for Community Healthcare, Hospital Development Institute Shanghai Jiao Tong University, Shanghai, China; ^4^ Department of General Surgery, Ren Ji Hospital, Shanghai Jiao Tong University School of Medicine, Shanghai, China

**Keywords:** mortality, older adults, cohort study, serum uric acid, gender difference

## Abstract

**Objectives:** This study aimed to prospectively investigate gender-specific relationship between hyperuricemia and all-cause mortality among Chinese older adults.

**Methods:** The study was based on the Chinese Longitudinal Healthy Longevity Survey (CLHLS) 2008–2018, a prospective nationwide cohort of older adults in China. Multivariate Cox proportional hazards models were used to estimate hazard ratios (HRs) and 95% CIs for all-cause mortality. Restricted cubic splines (RCS) were conducted to explore the dose-response relationship between SUA levels and all-cause mortality.

**Results:** For older women, compared to the participants in the third quartile of SUA level, those in the highest quartile of SUA was associated with significantly higher risk of all-cause mortality in the fully adjusted model (HR: 1.41, 95% CI: 1.03–1.92). No significant associations between SUA levels and all-cause mortality were observed in older men. The present study further found a U-shaped non-linear relationship between SUA levels and all-cause mortality in both sexes of older population (P for non-linear <0.05).

**Conclusions:** This study provided prospective epidemiological evidence for the predictive role of SUA on all-cause mortality among the Chinese aging population over 10 years of follow-up, while revealing considerable gender-related differences.

## Introduction

Serum uric acid (SUA), the final enzymatic product of purine metabolism, is a biological marker for the diagnosis of hyperuricemia. Increasing evidence linked hyperuricemia to a variety of adverse health outcomes, including hypertension (1, 2), obesity and diabetes (3, 4), dyslipidemia (5, 6), and kidney disease (7, 8), which all contributed to changes in all-cause mortality (9–11). However, epidemiological evidence on the relationship between hyperuricemia and all-cause mortality in older people remains inadequate and controversial. Several studies demonstrated that hyperuricemia was positively associated with all-cause mortality in the elder population (12–14). In contrast, few studies revealed that inverse associations between SUA levels and all-cause mortality resulting from SUA as a powerful antioxidant that has a protective effect on diseases (15). In addition, most of these studies were observational research that contained inevitable possible biases, thus whether hyperuricemia is an independent and causal risk factor for increased all-cause mortality, especially in Chinese older people remains unknown.

Higher SUA levels in men than in women at all ages have been widely demonstrated (16). Most previous studies on SUA levels and all-cause mortality only adjusted for sex in the models and seldom conducted sex-specific association analyses. Considering the gender differences in the distribution of SUA levels, some studies have analyzed the relationship between SUA levels and all-cause mortality, but the epidemiological evidence for the relationship in older adults is limited and inconsistent. On the one hand, several meta-analyses and observational studies have found that gender differences may modify the relationship between hyperuricemia and all-cause mortality, for example, only in males (17), females (18, 19) and/or both (12, 20). On the other hand, several meta-analyses and observational studies have shown no statistically significant gender differences in the association between SUA levels and all-cause mortality risk (21, 22). Most of the above contradictory results are based on observational studies and lack of evidence from prospective studies.

Considering those mentioned above, the aim of the present study was to examine the prospective associations between hyperuricemia and all-cause mortality in Chinese older adults over 10 years of follow-up. To our knowledge, few studies have examined the modified effect of gender on the longitudinal association between hyperuricemia and all-cause mortality among Chinese older adults. Therefore, this study further examined the gender differences in the relationship between hyperuricemia and all-cause mortality in the Chinese aging population.

## Methods

### Study Design and Participants

The CLHLS is the first and largest nationwide, community-based, longitudinal prospective cohort survey in China. The CLHLS were performed by randomly selected from counties or cities in 23 provinces across China, covering about 85% percent of the Chinese population (23). The CLHLS survey was initiated in 1998, and follow-up survey were conducted in 2000, 2002, 2005, 2008/2009, 2011/2012, 2014, and 2017/2018 (24). The CLHLS contained many types of information, including demographics, lifestyle, health status, and daily activities of older people, including a large percentage of the oldest population. Since biological indicator data were only available in the 2008–2009, 2011–2012, and 2014 waves, we therefore selected participants enrolled in 2008–2009 wave as our baseline population for follow-up. In 2009, a biomarker substudy was conducted in seven longevity regions, including Sanshui of Guangdong Province, Yongfu of Guangxi Autonomous Area, Chengmai of Hainan Province, Xiayi of Henan Province, Zhongxiang of Hubei Province, Mayang of Hunan Province, and Laizhou of Shandong Province. The CLHLS study was approved by the Research Ethics Committee of Peking University (IRB00001052-13074). Written informed consent was provided from all participants.

Initially, A total of 2035 participating older adults provided blood sample data in the 5th wave (2008–2009) of CLHLS. According to the following exclusion criteria, we retained 1,067 Chinese older adults in this study after applying the following exclusion criteria. 1) We excluded participants who had no participant ID and who were younger than 60 years old. 2) We excluded people with missing SUA and other biochemical data. 3) We excluded participants who were unable to match the 2008–2018 follow-up longitudinal survey. 4) We excluded participants due to their unknown information. The flow chart of the study population was shown in Supplementary Figure S1. The study population was divided into four groups according to quartiles of SUA levels by gender. The cutoff points were <241.30, 241.30–303.90, 303.90–355.80 and ≥355.80 μmol/L for males, and <201.65, 201.65–245.75, 245.75–301.70, and ≥301.70 μmol/L for females.

### Biochemical Measurements

Fasting venous blood samples were collected by trained medical personnel from all willing participant who had fasted overnight. These samples were collected into 5 mL heparin anticoagulant vacuum tubes and centrifuged at 20°C and 3000 × g for 10 min. The plasma was isolated and frozen at −20°C, shipped through cold-chained to the central laboratory at Capital Medical University in Beijing, and stored at −80°C until biochemical analysis. Plasma concentrations of blood urea nitrogen (BUN), creatinine, serum uric acid (SUA), fasting blood glucose, total cholesterol (TC), high-density lipoprotein cholesterol (HDL-C), low-density lipoprotein cholesterol (LDL-C) and triglyceride (TG) were measured using an automatic biochemistry analyzer (Hitachi 7180, Swiss Roche Company, Tokyo, Japan).

### Study Outcome During Prospective Follow-Up

The outcome of our study was all-cause mortality from 1 January 2008 to 31 December 2018. Mortality status was obtained from a publicly available dataset of the CLHLS, which captured the vital status of survey participants from baseline to 31 December 2018. If a participant missed the follow-up visit, the survival time was defined as the interval between baseline and time of missed visits. Survival time was calculated from the survey date at baseline until the date of death, or the interview date of follow-up survey, or lost visit date, whichever came first.

### Covariates

Covariates were collected information using a standardized questionnaire administered by the trained interviewers during an household interview. The present study adjusted for sociodemographic variables, health characteristics, and confounding biomarkers in the model, which were selected *a priori* based on previous studies examining risk factors for serum uric acid levels and all-cause mortality. The sociodemographic information included age, sex (male/female), residence (rural/other) and economic income (low/high). Health characteristics included lifestyle and history of chronic diseases. Lifestyle characteristics contained smoking (yes/no), drinking (yes/no), physical activity (current or former/none) and diet (frequency of consuming fresh fruit, vegetables, eggs, milk products, meat, fish and food made from beans). After the household interview, all participants were asked to undergo anthropometric measurements, which included systolic blood pressure (mmHg), diastolic blood pressure (mmHg) and body mass index (kg/m^2^). History of chronic disease including self-reported and/or hospital diagnosed hypertension, diabetes, heart disease, stroke or cerebrovascular disease and chronic nephritis. Blood biochemistry tests were analyzed in this study, included BUN (mmol/L), plasma creatinine (mmol/L), SUA (μmol/L), plasma glucose (mmol/L), TC (mmol/L), HDL-C (mmol/L), LDL-C (mmol/L), TG (mmol/L).

### Statistical Analysis

Continuous variables were presented as mean (standard deviation, SD) or median (interquartile range, IQR), and categorical variables were presented as counts (%).

For continuous variables that followed normal distribution, we used two independent sample t-test and ANOVA to compare the characteristics of participants in two and multiple groups, respectively. Tukey’s post-hoc test or Games-Howell post-hoc test was used to conduct pairwise comparisons for multiple groups. For the continuous variables with skewed distribution, Mann-Whitney U test and Kruskal-Wallis test with Dunn post-hoc test were used to compare the difference between two groups and multiple groups, respectively. Characteristics of the two and multiple groups were compared using chi-square test for categorical variables.

Multivariate Cox proportional hazards models were used to investigate relationships between SUA with all-cause mortality, using the third quartile (Q3) of SUA levels as a reference, and the results are presented as hazard ratios (HRs) and 95% confidence intervals (CIs). We performed analyses of the association between SUA and all-cause mortality by sex using four models based on previous studies as follows (8, 9, 25): Model 1 was crude; Model 2 was adjusted for age, category of residence, economic income, smoke, drink, physical activity and diet; Model 3 was further adjusted for SBP, DBP, BMI and history of chronic diseases; Model 4 was further adjusted for BUN, plasma creatinine, plasma glucose, TC, HDL cholesterol, LDL cholesterol and TG. Furthermore, the survival probability according to diagnostic values of hyperuricemia were calculated using the Kaplan-Meier curves, and the log-rank test was performed to analyze differences of all-cause mortality among quartiles of SUA. The multivariable Cox regression model’s discriminative performance was assessed by time-dependent receiver operating characteristic (ROC) curves and area under the curve (AUC) analysis.

In addition, subgroup analyses and their interactions were conducted to further explore whether gender would modify the relationship between SUA levels and all-cause mortality. Furthermore, multivariate Cox regression models with restricted cubic splines (RCS) methods were performed to explore the potentially non-linear relationship between SUA level and hazard ratio of all-cause mortality, using SUA level as a continuous variable. To balance best fit and overfitting in the main splines for all-cause mortality, the optimal model is selected based on the knot (ranging from 3 to 10) corresponding to the lowest value of the Akaike Information Criterion (AIC). In present study, we used restricted cubic splines methods with three knots to analyze the non-linear relationship.

Sensitivity analyses were performed to evaluate the robustness of our results: 1) using diagnostic values for clinically defined hyperuricemia by gender (male: 420 μmol/L; female: 360 μmol/L) instead of quartiles; 2) We excluded participants who were younger than 80 years old and further explored the relationship between SUA levels and all-cause mortality in the oldest-old adults.

The statistical analysis was performed using the R 4.0.3 Statistical Package (The R foundation for Statistical Computing). A two-sided *p* < 0.05 was considered to indicate statistical significance.

## Result

### Baseline Characteristics

Demographics, lifestyle, diet, history of chronic disease, and biochemical characteristics of all study participants were compared according to SUA level quartiles (Table 1). In brief, the mean age of study population was 84.84 years and 57.0% participants were female (*n* = 608). Participants who had higher SUA level tended to be older, lived in city or town areas, had higher income, drink alcohol, and eat meat and fish. In addition, participants with higher SUA levels had high levels of BUN, plasma creatinine, LDL-C and TG. The baseline characteristics of participants who remained in the cohort and were lost from the cohort due to lost to follow-up or death are summarized in Supplementary Tables S1, S2. The sex-specific baseline characteristics according to diagnostic values for hyperuricemia (male: 420 μmol/L; female: 360 μmol/L) are shown in Supplementary Table S3.

**TABLE 1:** Baseline characteristics of all study participants according to serum uric acid quartiles (Chinese Longitudinal Health Longevity Survey, China, 2008–2018).

Characteristics	All subjects	Serum uric acid quartiles (μmol/L)				*p*-value
	(N = 1,067)	Q1 (N = 266)	Q2 (N = 268)	Q3 (N = 266)	Q4 (N = 267)	
Demographic characteristics						
Age (years)	84.84 ± 13.15	81.23 ± 13.09	84.53 ± 13.59	86.00 ± 12.68	87.57 ± 12.45	**<0.001** ^e^ ^,^ ^f^
Female	608 (57.0)	152 (57.1)	152 (56.7)	151 (56.8)	153 (57.0)	0.999
Category of residence						**<0.001**
City/Town	245 (23.0)	40 (15.0)	54 (20.1)	66 (24.8)	85 (31.8)	
Rural	822 (77.0)	226 (85.0)	214 (79.9)	200 (75.2)	182 (68.2)	
Economic income (RMB/year)						**<0.001**
<10,000	509 (47.7)	161 (60.5)	125 (46.6)	115 (43.2)	108 (40.4)	
≥10,000	558 (52.3)	105 (39.5)	143 (53.4)	151 (56.8)	159 (59.6)	
Lifestyle						
Smoke						0.880
No	748 (70.1)	186 (69.9)	192 (71.6)	182 (68.4)	188 (70.4)	
Yes	319 (29.9)	80 (30.1)	76 (28.4)	84 (31.6)	79 (29.6)	
Drink						**0.030**
No	797 (74.7)	195 (73.3)	215 (80.2)	202 (75.9)	185 (69.3)	
Yes	270 (25.3)	71 (26.7)	53 (19.8)	64 (24.1)	82 (30.7)	
Physical activity						**<0.001**
Current/Former	280 (26.2)	45 (16.9)	69 (25.7)	79 (29.7)	87 (32.6)	
None	787 (73.8)	221 (83.1)	199 (74.3)	187 (70.3)	180 (67.4)	
Dietary						
Fresh fruit						**0.031**
Always/Often	379 (35.5)	77 (28.9)	98 (36.6)	94 (35.3)	110 (41.2)	
Sometimes/Rarely or never	688 (64.5)	189 (71.1)	170 (63.4)	172 (64.7)	157 (58.8)	
Fresh vegetables						0.333
Always/Often	974 (91.3)	244 (91.7)	238 (88.8)	248 (93.2)	244 (91.4)	
Sometimes/Rarely or never	93 (8.7)	22 (8.3)	30 (11.2)	18 (6.8)	23 (8.6)	
Eat Eggs						**0.002**
Always/Often	648 (60.7)	184 (69.2)	157 (58.6)	164 (61.7)	143 (53.6)	
Sometimes/Rarely or never	419 (39.3)	82 (30.8)	111 (41.4)	102 (38.3)	124 (46.4)	
Eat milk products						0.564
Always/Often	112 (10.5)	28 (10.5)	28 (10.4)	33 (12.4)	23 (8.6)	
Sometimes/Rarely or never	955 (89.5)	238 (89.5)	240 (89.6)	233 (87.6)	244 (91.4)	
Eat meat						**<0.001**
Always/Often	668 (62.6)	133 (50.0)	166 (61.9)	181 (68.0)	188 (70.4)	
Sometimes/Rarely or never	399 (37.4)	133 (50.0)	102 (38.1)	85 (32.0)	79 (29.6)	
Eat fish						**<0.001**
Always/Often	477 (44.7)	84 (31.6)	111 (41.4)	131 (49.2)	151 (56.6)	
Sometimes/Rarely or never	590 (55.3)	182 (68.4)	157 (58.6)	135 (50.8)	114 (43.4)	
Eat bean products						0.055
Always/Often	548 (51.4)	119 (44.7)	144 (53.7)	149 (56.0)	136 (50.9)	
Sometimes/Rarely or never	519 (48.6)	147 (55.3)	124 (46.3)	117 (44.0)	131 (49.1)	
Physical examination						
BMI (kg/m^2^)	20.22 ± 3.53	20.14 ± 3.24	20.28 ± 3.75	20.31 ± 3.50	20.17 ± 3.63	0.937
SBP (mmHg)	142.54 ± 22.04	142.89 ± 22.29	142.98 ± 21.95	143.08 ± 22.27	141.22 ± 21.72	0.732
DBP (mmHg)	78.68 ± 11.52	78.86 ± 11.66	78.45 ± 10.76	79.67 ± 11.34	77.76 ± 12.24	0.281
Biochemical parameters						
Blood Urea Nitrogen (mmol/L)^a^	6.67 ± 2.25	6.07 ± 1.60	6.25 ± 1.80	6.74 ± 2.11	7.63 ± 2.92	**<0.001** ^e^ ^,^ ^f^ ^,^ ^h^ ^,^ ^i^
Plasma creatinine (mmol/L)^b^	80.0 (66.0–100.0)	68.0 (55.0–79.0)	75.5 (62.0–92.0)	87.0 (74.0–105.3)	97.0 (81.0–125.0)	**<0.001** ^d–i^
Plasma glucose (mmol/L)	5.43 ± 1.89	5.28 ± 2.07	5.28 ± 1.67	5.52 ± 2.03	5.62 ± 1.75	0.089
Total cholesterol (mmol/L)^a^	3.49 ± 1.28	3.44 ± 1.04	3.48 ± 1.28	3.56 ± 1.34	3.48 ± 1.42	0.691
HDL cholesterol (mmol/L)	1.16 ± 0.32	1.14 ± 0.33	1.16 ± 0.30	1.16 ± 0.32	1.19 ± 0.33	0.441
LDL cholesterol (mmol/L)^a^	2.02 ± 0.77	1.81 ± 0.63	1.99 ± 0.75	2.11 ± 0.73	2.18 ± 0.91	**<0.001** ^e^ ^,^ ^f^
Triglyceride (mmol/L)^b^	1.08 (0.84–1.66)	0.94 (0.76–1.24)	1.09 (0.87–1.49)	1.19 (0.88–1.88)	1.20 (0.89–2.25)	**<0.001** ^d–f^ ^,^ ^h^
Disease History						
Hypertension	161 (15.1)	37 (13.8)	42 (15.7)	45 (16.9)	37 (14.0)	0.713
Diabetes mellitus	16 (1.5)	3 (1.1)	7 (2.6)	4 (1.5)	2 (0.8)	0.388
Heart diseases	63 (5.9)	23 (8.6)	13 (4.9)	16 (6.0)	11 (4.2)	0.128
Stroke or CVD	36 (3.4)	11 (4.1)	10 (3.7)	5 (1.9)	10 (3.8)	0.486
Cancer^c^	2 (0.2)	0 (0.0)	1 (0.4)	0 (0.0)	1 (0.4)	1.00
Chronic nephritis^c^	2 (0.2)	1 (0.4)	0 (0.0)	1 (0.4)	0 (0.0)	0.374

Abbreviations: SBP, systolic blood pressure; DBP, diastolic blood pressure; BMI, body mass index; HDL, high density lipoprotein; LDL, low density lipoprotein; CVD, cerebrovascular disease; Q1, 1st quartile; Q2, 2nd quartile; Q3, 3rd quartile; Q4, 4th quartile.

Data are presented as mean ± SD or mean (IQR) for continuous variables and n (%) for categorical variables.

^a^ Welch test due to uneven variance.

^b^ Kruskal-Wallis test.

^c^ Fisher’s exact probability test. ANOVA or Kruskal-Wallis test with *post hoc* test were performed to compare the potencial differences between quartiles of SUA level.

^d^ Statistical significance between Q1 level group and Q2 level group.

^e^ Statistical significance between Q1 level group and Q3 level group.

^f^ Statistical significance between Q1 level group and Q4 level group.

^g^ Statistical significance between Q2 level group and Q3 level group.

^h^ Statistical significance between Q2 level group and Q4 level group.

^i^ Statistical significance between Q3 level group and Q4 level group. Cut points for SUA quartile grouping being male: <241.30, 241.30–303.90, 303.90–355.80 and ≥355.80 μmol/L; female: <201.65, 201.65–245.75, 245.75–301.70 and ≥301.70 μmol/L.

Bold *p*-values indicated *p* < 0.05.

### Association of Serum Uric Acid and All-Cause Mortality

Table 2 presented the association between the level of SUA and all-cause mortality in all study population and sex-specific population. In the general population, the highest quartile (Q4) of SUA levels was associated with increased all-cause mortality in univariate model (Model 1: HR: 1.38; 95% CI: 1.10–1.73), when compared with the third quartile of SUA levels (Q3). This relationship remained statistically significant in the multivariate model (model 4) after adjusting for socio-demographic, health characteristics and clinical biochemical markers (HR: 1.34; 95% CI: 1.06–1.71). No statistically significant results were observed between SUA levels and all-cause mortality in men, neither in the crude model nor in the full-adjusted model. It was noteworthy that the significantly positive association between SUA levels and all-cause mortality still persisted in the female population. Compared with Q3 of SUA levels, the multivariate adjusted HR (model 4) for all-cause mortality was 1.41 (95% CI, 1.03–1.92) for Q4.

**TABLE 2:** Hazard ratios for all-cause mortality according to quartiles of serum uric acid in multivariate Cox regression analyses (Chinese Longitudinal Health Longevity Survey, China, 2008–2018).

Quartiles	Individuals	Events (%)	Person-years	Model 1		Model 2		Model 3		Model 4	
				HR (95%CI)	*p*-value	HR (95%CI)	*p*-value	HR (95%CI)	*p*-value	HR (95%CI)	*p*-value
All subjects											
Quartile 1	266	137 (51.12)	1545.58	0.90 (0.71–1.14)	0.388	1.16 (0.91–1.48)	0.237	1.15 (0.90–1.46)	0.279	1.08 (0.84–1.39)	0.557
Quartile 2	268	144 (53.73)	1440.33	1.03 (0.82–1.30)	0.809	1.12 (0.89–1.43)	0.333	1.14 (0.90–1.45)	0.273	1.12 (0.88–1.43)	0.358
Quartile 3	266	138 (51.88)	1415	References		References		References		References	
Quartile 4	267	159 (60.00)	1198.50	1.38 (1.10–1.73)	**0.006**	1.41 (1.12–1.78)	**0.004**	1.43 (1.13–1.80)	**0.003**	1.34 (1.06–1.71)	**0.016**
Males											
Quartile 1 (<241.30)	114	57 (50.00)	695.33	1.04 (0.71–1.51)	0.855	1.20 (0.79–1.81)	0.389	1.19 (0.77–1.83)	0.429	1.39 (0.89–2.17)	0.148
Quartile 2 (241.30–303.90)	116	50 (43.10)	708.67	0.89 (0.60–1.31)	0.547	1.01 (0.67–1.53)	0.970	1.09 (0.71–1.66)	0.701	1.24 (0.80–1.92)	0.330
Quartile 3 (303.90–355.80)	115	51 (44.35)	637.08	References		References		References		References	
Quartile 4 (>355.80)	114	58 (50.88)	575.25	1.26 (0.86–1.83)	0.231	1.29 (0.86–1.92)	0.221	1.30 (0.86–1.97)	0.219	1.34 (0.88–2.04)	0.176
Females											
Quartile 1 (<201.65)	152	80 (51.95)	850.25	0.82 (0.61–1.12)	0.208	1.13 (0.83–1.55)	0.435	1.11 (0.81–1.52)	0.516	1.02 (0.73–1.42)	0.916
Quartile 2 (201.65–245.75)	152	94 (61.84)	731.67	1.15 (0.86–1.54)	0.349	1.21 (0.90–1.63)	0.202	1.19 (0.88–1.60)	0.256	1.09 (0.81–1.49)	0.564
Quartile 3 (245.75–301.70)	151	87 (57.62)	777.92	References		References		References		References	
Quartile 4 (>301.70)	153	101 (66.89)	623.25	1.47 (1.11–1.96)	**0.008**	1.57 (1.17–2.10)	**0.003**	1.57 (1.17–2.11)	**0.003**	1.41 (1.03–1.92)	**0.033**

Abbreviations: HDL, high density lipoprotein; LDL-C, low density lipoprotein cholesterol; SBP, systolic blood pressure; DBP, diastolic blood pressure; BMI, body mass index; CVD, cerebrovascular disease. Model 1: crude model; Model 2: adjusted for age, sex, category of residence, economic income, smoke, drink, physical activity and dietary based on model 1; Model 3: further adjusted for SBP, DBP and BMI, self-reported hypertension, self-reported diabetes, self-reported heart disease, self-reported stroke or CVD and self-reported chronic nephritis based on model 2; Model 4: further adjusted for BUN, plasma creatinine, plasma glucose, TC, HDL-C, LDL-C and TG based on model 3; HR, Hazard ratio; CI, confidence interval.

Bold *p*-values indicated *p* < 0.05.

### Survival Analysis

**FIGURE 1:**
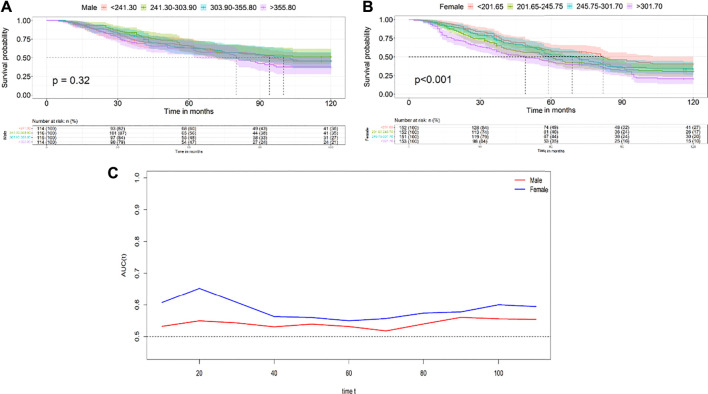
Kaplan-Meier survival survival curves for all-cause mortality according to quartiles of the serum uric acid levels in males **(A)** and females **(B)**; Time-dependent receiver operating characteristic analysis and areas under the curve of the multivariate Cox regression to predict all-cause mortality **(C)** (Chinese Longitudinal Health Longevity Survey, China, 2008–2018).

The Kaplan-Meier survival curves for all-cause mortality according to quartiles of serum uric acid are illustrated in Figure 1. In female participants, the survival probability between participants in each of the SUA quartiles show significantly difference (log-rank *p* < 0.001) (Figure 1B). However, in male participants, the survival probability between participants in each of the SUA quartiles did not show significantly difference (log-rank *p* = 0.320) (Figure 1A).

### Time-Dependent ROC Analysis of Predictive Accuracy for All-Cause Mortality

Time-dependent ROC analysis was conducted to compare the ability of the Model 4 to predict all-cause mortality between male and female. The AUC values of female in the total follow-up time were greater than those of male, which showed relatively good predictive accuracy in female (Figure 1C).

### Subgroup Analysis

**FIGURE 2:**
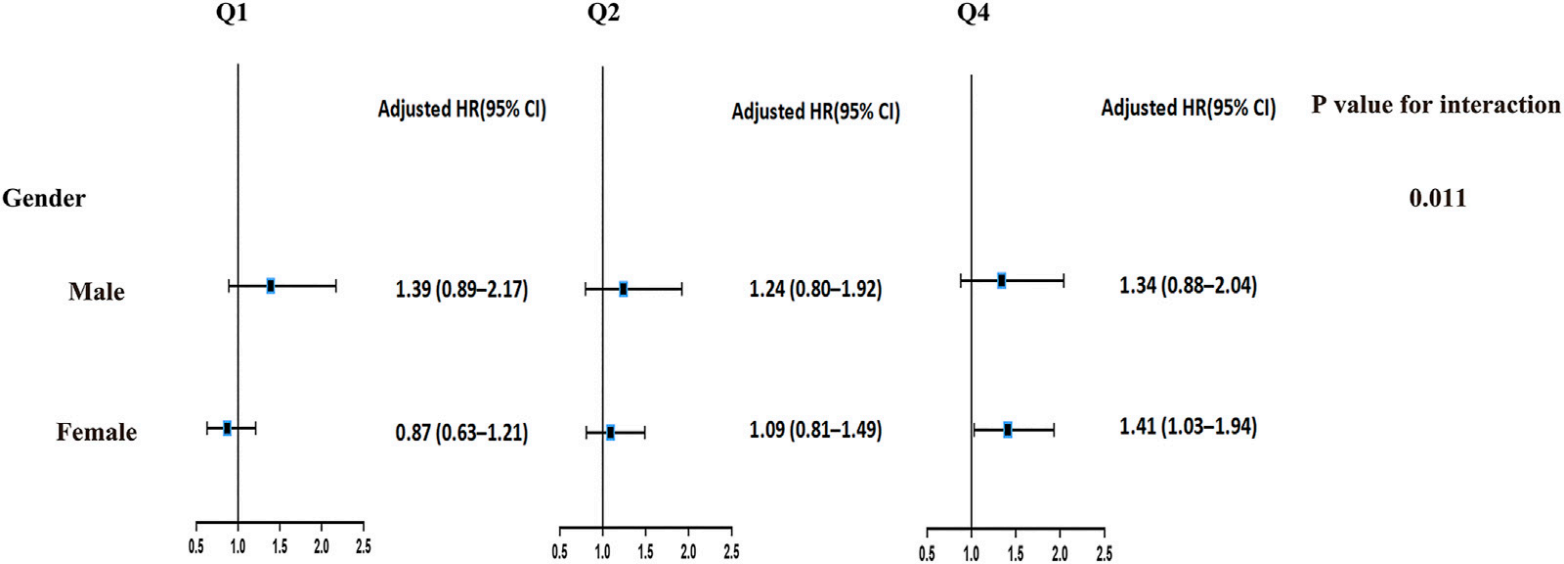
Subgroup analysis of the association between serum uric acid and risk of all-cause mortality (Chinese Longitudinal Health Longevity Survey, China, 2008–2018). Note: Hazard ratio (95% confidence interval) was calculated for Q1, Q2, and Q4 serum uric acid levels; Q3 serum uric acid level was taken as reference; Models were adjusted for significant confounding factors of age, residence, economic income, smoking, drinking, and physical activity; history of hypertension, diabetes mellitus, heart diseases, stroke or cerebrovascular diseases, and chronic nephritis; and blood urea nitrogen, high-density lipoprotein cholesterol, low-density lipoprotein cholesterol, triglyceride, total cholesterol, plasma creatinine and plasma glucose. Abbreviations: Q1, 1st quartile; Q2, 2nd quartile; Q3, 3rd quartile; Q4, 4th quartile; HR, Hazard ratio; CI, confidence interval.

Subgroup analysis showed that the interaction *p*-value for gender was 0.011, which indicated that the association between SUA levels and all-cause mortality was significantly different by gender after adjusting for a range of covariates (Figure 2).

### A Non-Linear Relationship Between Serum Uric Acid and All-Cause Mortality

**FIGURE 3:**
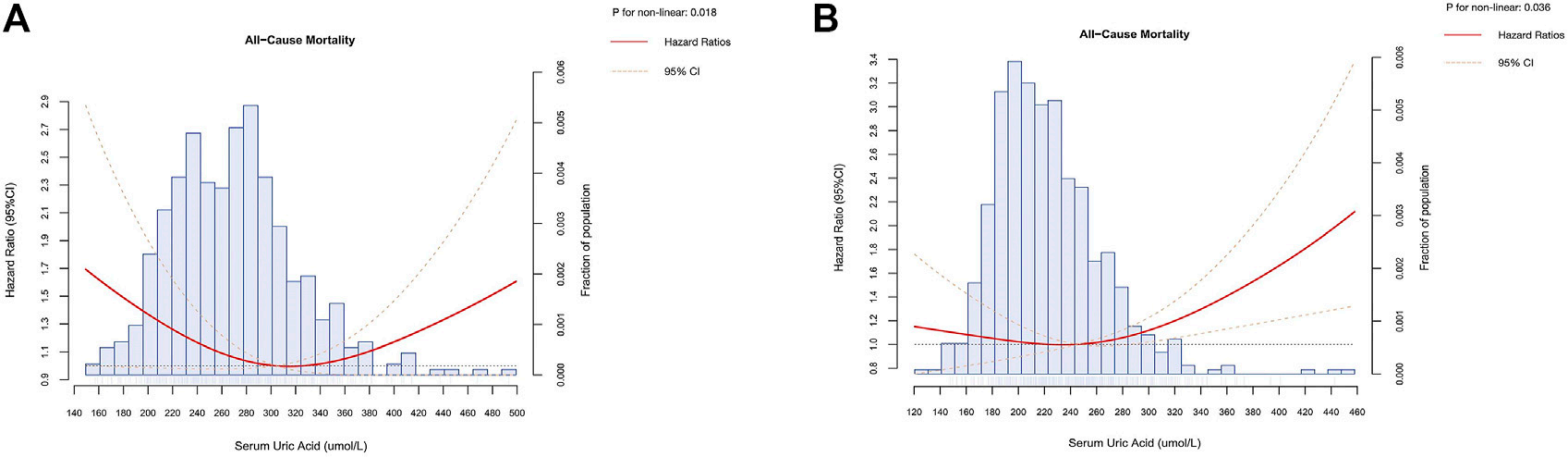
Dose-response association of serum uric acid with all-cause mortality in male **(A)** and female **(B)** (Chinese Longitudinal Health Longevity Survey, China, 2008–2018). Note: Hazard ratio (solid red line) and 95% confidence interval (dashed orange lines) were obtained from multivariate Cox regression models with restricted cubic splines methods. Reference lines for no association were indicated by the dashed black lines at a hazard ratio of 1.00. Multivariable adjustment was performed for sociodemographics (age, category of residence and income), health characteristics (smoking, drinking, physical activity and diet, systolic blood pressure, diastolic blood pressure, body mass index, history of hypertension, diabetes mellitus, heart diseases, stroke or cerebrovascular diseases and chronic nephritis) and confounding biomarkers (plasma creatinine, plasma glucose, blood urea nitrogen, high-density lipoprotein cholesterol , low-density lipoprotein cholesterol, triglyceride and total cholesterol).

To further evaluate a possible non-linear relationship, restricted cubic spline was further performed to investigate the association between all-cause mortality and SUA on a continuous scale. Consistent with the results shown in Table 2, the multivariate Cox regression models with restricted cubic splines methods indicated that the relationship between serum uric acid with all-cause mortality was U shaped both in male and female (*P* for non-linear<0.05), and the lowest risk was observed to be within the third quartile SUA levels for men and women, respectively (Figure 3). Low and high levels of SUA were associated with an increased risk of all-cause mortality.

### Sensitivity Analysis

The relationship between serum SUA levels and all-cause mortality remained consistent in sensitivity analyses after using clinically defined cut-off values for SUA levels (Supplementary Table S4). In female participants, compared to the participants in lower than 360 μmol/L of serum uric acid, those in higher than 360 μmol/L of serum uric acid were associated with increased all-cause mortality in full-adjusted model (Model 4: HR, 1.65; 95% CI, 1.13–2.41). However, in male participants, compared to the participants in lower than 420 μmol/L of serum uric acid, those in higher than 420 μmol/L of serum uric acid were not associated with increased all-cause mortality in univariable and multivariate model. The KM survival curves and time-dependent ROC analysis of all-cause mortality according to the SUA clinical diagnostic reference value (male: 420 μmol/L; female: 360 μmol/L) are shown in Supplementary Figures S2, S3, which also showed similar results. In addition, it is noteworthy that the highest quartile of SUA levels (Q4) was also found to be associated with an increased risk of all-cause mortality compared to the reference SUA levels (Q3) in the study population confined to oldest-old adults. Meanwhile, the lowest quartile of SUA levels (Q1) was associated with an increased risk of all-cause mortality, which might also confirm that the U-shaped relationship between SUA levels and all-cause mortality risk also persisted in the oldest-old subjects (Supplementary Table S5).

## Discussion

This study revealed a potential causal association between hyperuricemia and 10-year all-cause mortality in the Chinese aging population. Our study in the aging population also supported the common consensus that hyperuricemia might increase all-cause mortality. It was noteworthy that this finding was gender-specific, and the association was particularly pronounced among the female older population. In addition, the present study observed non-linear associations between SUA levels and all-cause mortality in both older males and females.

To date, current epidemiological studies on the relationship between SUA levels and all-cause mortality among older adults have been rarely investigated and available evidence is inconsistent. Several studies provided evidence to support the predictive value and risk effects of hyperuricemia or higher SUA levels on all-cause mortality in the aging population (12, 13, 26). Conversely, few previous studies failed to confirm the associations between hyperuricemia and all-cause mortality in the aging population (21, 27). These controversial findings might be induced partially by the differences in the type of study, the characteristics of the study population (i.e., gender, ethnicity, and study region), sample size, cutoff points of hyperuricemia, and adjustment of confounders (physical activity, diet, history of chronic diseases, blood biochemical indicators and so on). Therefore, we used stepwise models to adjust for various possible confounders. By extensively adjusting for potential confounders and performing gender-stratified analysis, our study provided prospective evidence that hyperuricemia might independently predict all-cause mortality in Chinese older adults.

Consistent with previous observations in the general population, the older men in the present study had significantly higher levels of SUA than the older women, implying that the relationship with all-cause mortality was complex and strongly related to gender. However, there were no definite conclusions about gender differences between hyperuricemia and all-cause mortality, which partly due to differences in the study population. Some previous observations in general adults found that higher SUA levels are more strongly associated with all-cause mortality in women than in men (28, 29). Nevertheless, an observational study using NHANES data in US adults found that high uric acid levels were associated with increased all-cause mortality regardless of gender, with a higher risk of all-cause mortality in men compared to women (30). However, few studies in healthy adults observed the association of SUA levels with all-cause mortality among men, but not women (31). These inconsistent findings also implied that it is necessary to stratify by gender when investigating the relationship between hyperuricemia and all-cause mortality in older adults.

Most noteworthy, when the all-cause mortality risk was analyzed according to quartiles of uric acid levels, our study demonstrated that the significant association between hyperuricemia and all-cause mortality was only in the female older population, which was consistent with some previous findings in the older population (14, 18, 19). Similar results were found in sensitivity analyses by using clinical cutoffs of dichotomous SUA levels. In addition, there were no statistically significant associations between SUA levels and all-cause mortality in both men and women after adjusting for potential confounders in the recent aged physical examination cohort from Shanghai, China (21). Therefore, the non-significant relationship found in older men in this study also seemed not abnormal. This finding might be attributed to the older age of the study population and the lower proportion of males with hyperuricemia in this study. In addition, most studies had shown that males with hyperuricemia or abnormal uric acid levels developed diseases such as kidney and liver function injury concentrated in the age range from 40 to 60 years, and then increase the risk of disease-related mortality and all-cause mortality (32–36). These reasons might potentially explain the non-significant association of hyperuricemia in older males on all-cause mortality that contradicted previous literature published in the general population. The current findings not only extend the scope of the study population exploring the relationship between hyperuricemia and all-cause mortality, but also provide new longitudinal evidence for exploring these associations in Chinese older adults. Therefore, future preventive measures related to hyperuricemia should be targeted towards older women as the susceptible population.

Several potential mechanisms could explain the modified role of gender on the relationship between hyperuricemia and all-cause mortality in the aging population. Estrogen levels and the complex metabolic changes associated with menopause might partially explain the gender differences. Estrogens that promote renal excretion of uric acid may keep serum uric acid levels low in women (37). However, one study showed that the level of estrogen that protects against the development of atherosclerosis in postmenopausal women is about half that of men, so hyperuricemia in older women might increase the risk of all-cause mortality (38, 39). Menopause has recently been associated with significant increases in SUA levels, implicating the increased risk of hyperuricemia in older women (28, 40). Therefore, our findings could be explained by the hypothesis that postmenopausal women are more susceptible to hyperuricemia than men, and accordingly all-cause mortality is significantly higher in older women than in older men due to the putative pro-oxidant, pro-inflammatory effects of hyperuricemia. The mechanism for hyperuricemia on the increased risk of all-cause mortality in postmenopausal women still need to be further explored. Another potential explanation might be that the LDL cholesterol and total cholesterol levels in women were higher than in men in the current study. The positive relationship between LDL and total cholesterol and hyperuricemia has been studied, especially in women, which is in line with the characteristic profile of higher SUA levels group by gender in this study (41–43).

Furthermore, we found the U-shaped associations between SUA levels and all-cause mortality were observed in both older men and women when SUA levels were analyzed as a continuous variable. A population-based cohort study found a U-shaped association between SUA levels and all-cause mortality risk in the older population aged >65 years in Taiwan, China (44). However, the above studies were not analyzed stratified by gender, which may modify the association between SUA levels and mortality due to gender differences in the distribution of SUA levels. Wu et al. observed a U-shaped non-linear association between SUA levels and all-cause mortality in older men, in agreement with the present study, but failed to provide evidence of a non-linear relationship among older women (14). The above differences of non-linear relationship between SUA levels and all-cause mortality risk in the older population depend on the SUA levels of the study population and the sex-specific thresholds for classifying hyperuricemia. This study also provides new evidence to explore the non-linear relationship between SUA levels and all-cause mortality risk in the aging population. In addition, the present study found that whenever SUA levels were analyzed as quartiles or continuous variables, the lowest hazard ratios for all-cause mortality were found for SUA levels in the third quartile, which is below the current clinical diagnostic criteria for SUA levels. Therefore, this study highlighted that the normal or optimal level of SUA in older adults should be moderately adjusted in the context of clinical practice. These findings might provide the theoretical evidence and clinical guidelines on primary prevention of hyperuricemia and reducing the burden of various diseases caused by hyperuricemia in older people.

This study has several strengths that overcome some of the limitations of previous studies. Our study collected a Chinese elderly cohort with a long follow-up period covering 7 provinces, which could provide more representative longitudinal evidence on the association between hyperuricemia and all-cause mortality in older adults. Additionally, our study employed gender-stratified analysis to help understand the gender differences between hyperuricemia and all-cause mortality in the Chinese older population. However, several limitations of the present study also should be mentioned. First, the sample size of the older population followed in this study was relatively small because biological sample data of the Chinese oldest old in the CLHLS database were not easily available. We hope that future large population-based cohort studies and experimental studies will further explore the gender-specific associations between hyperuricemia and all-cause mortality in older adults and reveal the underlying mechanisms involved. Second, the population of this study included Chinese older adults, so the findings may be limited by extrapolating to the general population of China and other countries and ethnic. Thirdly, information on the medication use of hyperuricemia and other medications (i.e., diuretics) in the study population was not available during baseline information collection, which might influence the relationship between hyperuricemia and all-cause mortality. Finally, exploration of the potential relationship between serum uric acid levels and cause-specific mortality could not be performed because of the unavailable cause-specific mortality data.

### Conclusion

In summary, the relationship between SUA levels and all-cause mortality was modified by gender among Chinese older adults. This longitudinal finding of 10-year follow-up was pronounced in older women participants, but not in older men participants. In addition, our study provided the new epidemiologic evidence that the U-shaped non-linear relationship between SUA levels and all-cause mortality was present across gender among Chinese older adults. Further studies are needed to elucidate the actual role of SUA in all-cause mortality among older adults as well as potential biological mechanisms.
