# Supplementary material for: Gender-Specific and U-Shaped Relationship Between Serum Uric Acid and All-Cause Mortality Among Chinese Older Adults: A National Population-Based Longitudinal Study
Source: Int J Public Health. 2023 May 3;68:1605934. doi: 10.3389/ijph.2023.1605934 (PMC10188991; doi:10.3389/ijph.2023.1605934)
Supplement: Supplementary file 1 [file DataSheet1.docx]

**Gender-specific and U-shaped relationship between serum uric acid and all-cause mortality among Chinese older adults: the Chinese Longitudinal Healthy Longevity Survey 2008-2018**

**SUPPLEMENTARY DATA**

Table of Contents

[1. Supplementary Figure 1. Flow chart on the selection of study population. 2](#_Toc127883287)

[2. Supplementary Table 1. Baseline characteristics of the study population in male. 3](#_Toc127883288)

[3. Supplementary Table 2. Baseline characteristics of the study population in female. 5](#_Toc127883289)

[4. Supplementary Table 3 Baseline characteristics of participants based on clinical hyperuricemia diagnostic criteria by gender (sensitivity analysis) 8](#_Toc127883290)

[5. Supplementary Table 4 Association of hyperuricemia with 10-year all-cause mortality by gender among older adults in China (sensitivity analysis) 11](#_Toc127883291)

[6. Supplementary Figure 2 Kaplan-Meier survival curves for all-cause mortality categorized by clinical hyperuricemia diagnostic criteria in men (A) and women (B) (sensitivity analysis) 12](#_Toc127883292)

[7. Supplementary Figure 3. Time-dependent ROC analysis and areas under the curve of the multivariate Cox regression to predict all-cause mortality (sensitivity analysis) 14](#_Toc127883293)

[8. Supplementary Table 5. Hazard ratios for all-cause mortality among subjects aged≧80 years old in multivariate Cox regression analysis (N=704) (sensitivity analysis) 15](#_Toc127883294)

# Supplementary Figure 1. Flow chart on the selection of study population.


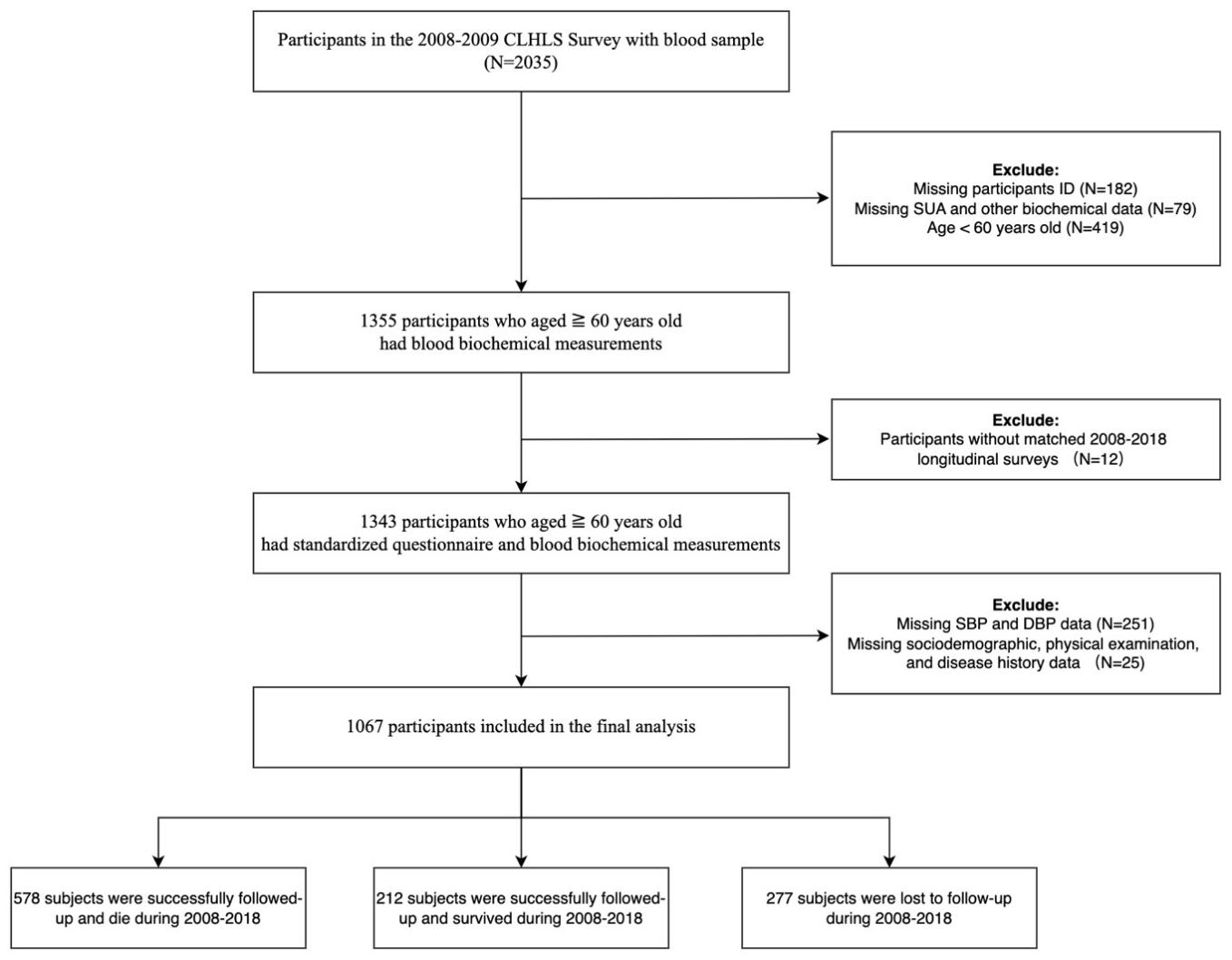


# Supplementary Table 1. Baseline characteristics of the study population in male.

| **Characteristics** | **Total (n=459)** | **Survivors(n=118)** | **Death(n=216)** | **Lost to follow-up(n=125)** |
| --- | --- | --- | --- | --- |
| Age (years) |  |  |  |  |
| <80 | 226 (49.24) | 92 (77.97) | 52 (24.07) | 82 (65.60) |
| ≥80 | 233 (50.76) | 26 (22.03) | 164 (75.93) | 43 (34.40) |
| Area of residence |  |  |  |  |
| City/Town | 119 (25.93) | 24 (20.34) | 42 (19.44) | 53 (42.40) |
| Rural | 340 (74.07) | 94 (79.66) | 174 (80.56) | 72 (57.60) |
| Economic income (RMB/year) |  |  |  |  |
| <10000 | 227 (49.46) | 59 (50.00) | 120 (55.56) | 48 (38.40) |
| ≥10000 | 232 (50.54) | 59 (50.00) | 96 (44.44) | 77 (61.60) |
| Smoke |  |  |  |  |
| No | 192 (41.83) | 52 (44.07) | 90 (41.67) | 50 (40.00) |
| Yes | 267 (58.17) | 66 (55.93) | 126 (58.33) | 75 (60.00) |
| Drink |  |  |  |  |
| No | 261 (56.86) | 61 (51.69) | 128 (59.26) | 72 (57.60) |
| Yes | 198 (43.14) | 57 (48.31) | 88 (40.74) | 53 (42.40) |
| Physical activity |  |  |  |  |
| Current/Former | 136 (29.63) | 36 (30.51) | 60 (27.78) | 40 (32.00) |
| None | 323 (70.37) | 82 (69.49) | 156 (72.22) | 85 (68.00) |
| Fresh fruit |  |  |  |  |
| Always/Often | 171 (37.25) | 55 (46.61) | 67 (31.02) | 49 (39.20) |
| Sometimes/Rarely or never | 288 (62.75) | 63 (53.39) | 149 (68.98) | 76 (60.80) |
| Fresh vegetables |  |  |  |  |
| Always/Often | 423 (92.16) | 111 (94.07) | 195 (90.28) | 117 (93.60) |
| Sometimes/Rarely or never | 36 (7.84) | 7 (5.93) | 21 (9.72) | 8 (6.40) |
| Eat Eggs |  |  |  |  |
| Always/Often | 291 (63.40) | 92 (77.97) | 132 (61.11) | 67 (53.60) |
| Sometimes/Rarely or never | 168 (36.60) | 26 (22.03) | 84 (38.89) | 58 (46.40) |
| Eat milk products |  |  |  |  |
| Always/Often | 40 (8.71) | 11 (9.32) | 21 (9.72) | 8 (6.40) |
| Sometimes/Rarely or never | 419 (91.29) | 107 (90.68) | 195 (90.28) | 117 (93.60) |
| Eat meat |  |  |  |  |
| Always/Often | 291 (63.40) | 78 (66.10) | 130 (60.19) | 83 (66.40) |
| Sometimes/Rarely or never | 168 (36.60) | 40 (33.90) | 86 (39.81) | 42 (33.60) |
| Eat fish |  |  |  |  |
| Always/Often | 214 (46.62) | 57 (48.31) | 88 (40.74) | 69 (55.20) |
| Sometimes/Rarely or never | 245 (53.38) | 61 (51.69) | 128 (59.26) | 56 (44.80) |
| Eat bean products |  |  |  |  |
| Always/Often | 226 (29.24) | 68 (57.63) | 106 (49.07) | 52 (41.60) |
| Sometimes/Rarely or never | 233 (50.76) | 50 (42.37) | 110 (50.93) | 73 (58.40) |
| SBP (mmHg) | 141.67±21.02 | 138.64±19.93 | 144.92±21.03 | 138.90±21.35 |
| DBP (mmHg) | 78.91±11.56 | 77.61±11.51 | 79.57±12.20 | 79.00±10.41 |
| BMI (kg/m^2^) | 21.03±3.28 | 21.58±3.42 | 20.45±2.97 | 21.50±3.50 |
| Blood Urea Nitrogen (mmol/L) | 6.60±1.98 | 6.24±1.75 | 6.97±2.11 | 6.31±1.84 |
| Plasma creatine (mmol/L) | 89.00 (76.00-110.00) | 83.00 (73.25-99.75) | 92.00 (74.75-117.00) | 93.00 (79.00-105.00) |
| Serum uric acid (μmol/L) | 305.97±88.65 | 287.65±81.35 | 309.00±94.44 | 318.03±82.69 |
| Plasma glucose (mmol/L) | 5.50±2.00 | 5.28±1.79 | 5.48±2.03 | 5.74±2.12 |
| Total cholesterol (mmol/L) | 3.14±1.23 | 3.24±1.19 | 3.30±1.12 | 2.75±1.38 |
| HDL cholesterol (mmol/L) | 1.10±0.31 | 1.04±0.33 | 1.12±0.30 | 1.13±0.31 |
| LDL cholesterol (mmol/L) | 1.87±0.74 | 1.84±0.70 | 1.80±0.70 | 2.03±0.84 |
| Triglyceride (mmol/L) | 1.03 (0.79-1.67) | 0.79 (1.02-1.61) | 0.95 (0.75-1.29) | 1.27 (0.88-3.16) |
| Hypertension | 55 (11.98) | 15 (12.71) | 27 (12.50) | 13 (10.40) |
| Diabetes mellitus | 8 (1.74) | 2 (1.69) | 4 (1.85) | 2 (1.60) |
| Heart diseases | 23 (5.01) | 3 (2.54) | 14 (6.48) | 6 (4.80) |
| Stroke or CVD | 21 (4.58) | 3 (2.54) | 15 (6.94) | 3 (2.40) |
| Cancer | 1 (0.22) | 0 (0.00) | 1 (0.46) | 0 (0.00) |
| Chronic nephritis | 1 (0.22) | 0 (0.00) | 1 (0.46) | 0 (0.00) |
| **Abbreviations:** SBP, systolic blood pressure; DBP, diastolic blood pressure; BMI, body mass index; HDL, high density lipoprotein; LDL, low density lipoprotein; CVD, cerebrovascular disease. Data are presented as mean ± SD or Median (IQR) for continuous variables and n (%) for categorical variables. | | | | |

# Supplementary Table 2. Baseline characteristics of the study population in female.

| **Characteristics** | **Total(n=608)** | **Survivors(n=94)** | **Death(n=362)** | **Lost to follow-up(n=152)** | |
| --- | --- | --- | --- | --- | --- |
| Age (years) |  |  |  |  |  |
| <80 | 137 (22.53) | 53 (56.38) | 24 (6.63) | 60 (39.47) |  |
| ≥80 | 471 (77.47) | 41 (43.62) | 338 (93.37) | 92 (60.53) |  |
| Area of residence |  |  |  |  |  |
| City/Town | 126 (20.72) | 16 (17.02) | 60 (16.57) | 50 (32.89) |  |
| Rural | 482 (79.28) | 78 (82.98) | 302 (83.43) | 102 (67.11) |  |
| Economic income (RMB/year) |  |  |  |  |  |
| <10000 | 282 (46.38) | 43 (45.74) | 181 (50.00) | 58 (38.16) |  |
| ≥10000 | 326 (53.62) | 51 (54.26) | 181 (50.00) | 94 (61.84) |  |
| Smoke |  |  |  |  |  |
| No | 556 (91.45) | 87 (92.55) | 330 (91.16) | 139 (91.45) |  |
| Yes | 52 (8.55) | 7 (7.45) | 32 (8.84) | 13 (8.55) |  |
| Drink |  |  |  |  |  |
| No | 536 (88.16) | 85 (90.43) | 318 (87.85) | 133 (87.50) |  |
| Yes | 72 (11.84) | 9 (9.57) | 44 (12.15) | 19 (12.50) |  |
| Physical activity |  |  |  |  |  |
| Current/Former | 144 (23.68) | 20 (21.28) | 87 (24.03) | 37 (24.34) |  |
| None | 464 (76.32) | 74 (78.72) | 275 (75.97) | 115 (75.66) |  |
| Fresh fruit |  |  |  |  |  |
| Always/Often | 208 (34.21) | 36 (38.30) | 122 (33.70) | 50 (32.89) |  |
| Sometimes/Rarely or never | 400 (65.79) | 58 (61.70) | 240 (66.30) | 102 (67.11) |  |
| Fresh vegetables |  |  |  |  |  |
| Always/Often | 551 (90.62) | 89 (94.68) | 325 (89.78) | 137 (90.13) |  |
| Sometimes/Rarely or never | 57 (9.38) | 5 (5.32) | 37 (10.22) | 15 (9.87) |  |
| Eat Eggs |  |  |  |  |  |
| Always/Often | 357 (58.72) | 65 (69.15) | 215 (59.39) | 77 (50.66) |  |
| Sometimes/Rarely or never | 251 (41.28) | 29 (30.85) | 147 (40.61) | 75 (49.34) |  |
| Eat milk products |  |  |  |  |  |
| Always/Often | 72 (11.84) | 6 (6.38) | 52 (14.36) | 14 (9.21) |  |
| Sometimes/Rarely or never | 536 (88.16) | 88 (93.62) | 310 (85.64) | 138 (90.79) |  |
| Eat meat |  |  |  |  |  |
| Always/Often | 377 (62.01) | 52 (55.32) | 227 (62.71) | 98 (64.47) |  |
| Sometimes/Rarely or never | 231 (37.99) | 42 (44.68) | 135 (37.29) | 54 (35.53) |  |
| Eat fish |  |  |  |  |  |
| Always/Often | 263 (43.26) | 36 (38.30) | 154 (42.54) | 73 (48.03) |  |
| Sometimes/Rarely or never | 345 (56.74) | 58 (61.70) | 208 (57.46) | 79 (51.97) |  |
| Eat bean products |  |  |  |  |  |
| Always/Often | 322 (52.96) | 52 (55.32) | 204 (56.35) | 66 (43.42) |  |
| Sometimes/Rarely or never | 286 (47.04) | 42 (44.68) | 158 (43.65) | 86 (56.58) |  |
| DBP (mmHg) | 143.20±22.77 | 143.96±23.31 | 143.24±23.75 | 142.64±20.03 |  |
| DBP (mmHg) | 78.51±11.49 | 80.23±11.09 | 77.81±11.91 | 79.13±10.59 |  |
| BMI (kg/m^2^) | 19.62±3.60 | 20.37±3.39 | 19.22±3.85 | 20.08±2.94 |  |
| Blood Urea Nitrogen (mmol/L) | 6.72±2.44 | 5.94±1.76 | 6.97±2.49 | 6.61±2.56 |  |
| Plasma creatine (mmol/L) | 73.50 (60.00-92.75) | 69.5 (58.25-81.00) | 60.0 (75.00-94.75) | 61.00 (75.00-96.00) |  |
| Serum uric acid (μmol/L) | 258.63±79.94 | 232.56±65.03 | 266.65±84.42 | 255.66±73.91 |  |
| Plasma glucose (mmol/L) | 5.37±1.81 | 5.20±1.89 | 5.42±1.66 | 5.36±2.08 |  |
| Total cholesterol (mmol/L) | 3.76±1.25 | 3.61±1.28 | 3.89±1.11 | 3.53±1.46 |  |
| HDL cholesterol (mmol/L) | 1.20±0.32 | 1.10±0.36 | 1.24±0.31 | 1.19±0.29 |  |
| LDL cholesterol (mmol/L) | 2.14±0.78 | 2.09±0.70 | 2.11±0.76 | 2.24±0.85 |  |
| Triglyceride (mmol/L) | 1.11 (0.88-1.65) | 1.19 (0.97-1.93) | 0.86 (1.05-1.37) | 1.26 (0.93-2.39) |  |
| Hypertension | 106 (17.43) | 16 (17.02) | 59 (16.30) | 31 (20.39) |  |
| Diabetes mellitus | 8 (1.32) | 3 (3.19) | 2 (0.55) | 3 (1.97) |  |
| Heart diseases | 40 (6.58) | 11 (11.70) | 17 (4.70) | 12 (7.89) |  |
| Stroke or CVD | 15 (2.47) | 3 (3.19) | 9 (2.49) | 3 (1.97) |  |
| Cancer | 1 (0.16) | 0 (0.00) | 0 (0.00) | 1 (0.66) |  |
| Chronic nephritis | 1 (0.16) | 0 (0.00) | 1 (0.28) | 0 (0.00) |  |
| **Abbreviations:** SBP, systolic blood pressure; DBP, diastolic blood pressure; BMI, body mass index; HDL, high density lipoprotein; LDL, low density lipoprotein; CVD, cerebrovascular disease. Data are presented as mean ± SD or mean (IQR) for continuous variables and n (%) for categorical variables. | | | | |  |

# Supplementary Table 3 Baseline characteristics of participants based on clinical hyperuricemia diagnostic criteria by gender (sensitivity analysis)

| **Characteristics** | **Male** | | |  | **Female** | | |
| --- | --- | --- | --- | --- | --- | --- | --- |
|  | **SUA≤420μmol/L**  **n=415** | **SUA>420μmol/L**  **n=44** | ***P*-value** |  | **SUA≤360μmol/L**  **n=547** | **SUA>360μmol/L**  **n=61** | ***P*-value** |
| **Demographic characteristics** |  |  |  |  |  |  |  |
| Age (years) |  |  | 0.245 |  |  |  | **0.029** |
| <80 | 208 (50.12) | 18 (40.91) |  |  | 130 (23.77) | 7 (11.48) |  |
| ≥80 | 207 (49.88) | 26 (59.09) |  |  | 417 (76.23) | 54 (88.52) |  |
| Area of residence |  |  | 0.097 |  |  |  | **0.001** |
| City/Town | 103 (24.82) | 16 (36.36) |  |  | 103 (18.83) | 23 (37.70) |  |
| Rural | 312 (75.18) | 28 (63.64) |  |  | 444 (81.17) | 38 (62.30) |  |
| Economic income (RMB/year) |  |  | **0.032** |  |  |  | **0.048** |
| <10000 | 212 (51.08) | 15 (34.09) |  |  | 261 (47.71) | 21 (34.43) |  |
| ≥10000 | 203 (48.92) | 29 (65.91) |  |  | 286 (52.29) | 40 (65.57) |  |
| **Lifestyle** |  |  |  |  |  |  |  |
| Smoke |  |  | 0.896 |  |  |  | 0.179 |
| No | 174 (41.93) | 18 (40.91) |  |  | 503 (91.96) | 53 (86.89) |  |
| Yes | 241 (58.07) | 26 (59.09) |  |  | 44 (8.04) | 8 (13.11) |  |
| Drink |  |  | 0.198 |  |  |  | 0.746 |
| No | 240 (57.83) | 21 (47.73) |  |  | 483 (88.30) | 53 (86.89) |  |
| Yes | 175 (42.17) | 23 (52.27) |  |  | 64 (11.70) | 8 (13.11) |  |
| Physical activity |  |  | 0.085 |  |  |  | 0.078 |
| Current/Former | 118 (28.4) | 18 (40.9) |  |  | 124 (22.7) | 20 (32.8) |  |
| None | 297 (71.6) | 26 (59.1) |  |  | 423 (77.3) | 41 (67.2) |  |
| **Dietary** |  |  |  |  |  |  |  |
| **Low purine foods** |  |  |  |  |  |  |  |
| Fresh fruit |  |  | 0.131 |  |  |  | 0.373 |
| Always/Often | 150 (36.14) | 21 (47.73) |  |  | 184 (33.64) | 24 (39.34) |  |
| Sometimes/Rarely or never | 265 (63.86) | 23 (52.27) |  |  | 363 (66.36) | 37 (60.66) |  |
| Fresh vegetables |  |  | 0.072 |  |  |  | 0.896 |
| Always/Often | 386 (93.01) | 37 (84.09) |  |  | 496 (90.68) | 55 (90.16) |  |
| Sometimes/Rarely or never | 29 (6.99) | 7 (15.91) |  |  | 51 (9.32) | 6 (9.84) |  |
| Eat Eggs |  |  | **0.023** |  |  |  | 0.111 |
| Always/Often | 270 (65.06) | 21 (47.73) |  |  | 327 (59.78) | 30 (49.18) |  |
| Sometimes/Rarely or never | 145 (34.94) | 23 (52.27) |  |  | 220 (40.22) | 31 (50.82) |  |
| Eat milk products |  |  | 0.851 |  |  |  | 0.926 |
| Always/Often | 37 (8.92) | 3 (6.82) |  |  | 65 (11.88) | 7 (11.48) |  |
| Sometimes/Rarely or never | 378 (91.08) | 41 (93.18) |  |  | 482 (88.12) | 54 (88.52) |  |
| **High purine foods** |  |  |  |  |  |  |  |
| Eat meat |  |  | 0.177 |  |  |  | **0.011** |
| Always/Often | 259 (62.41) | 32 (72.73) |  |  | 330 (60.33) | 47 (77.05) |  |
| Sometimes/Rarely or never | 156 (37.59) | 12 (27.27) |  |  | 217 (39.67) | 14 (22.95) |  |
| Eat fish |  |  | **<0.001** |  |  |  | **0.038** |
| Always/Often | 182 (43.86) | 32 (72.73) |  |  | 229 (41.86) | 34 (55.74) |  |
| Sometimes/Rarely or never | 233 (56.14) | 12 (27.27) |  |  | 318 (58.14) | 27 (44.26) |  |
| Eat bean products |  |  | 0.915 |  |  |  | 0.088 |
| Always/Often | 204 (49.16) | 22 (50.00) |  |  | 296 (54.11) | 26 (42.62) |  |
| Sometimes/Rarely or never | 211 (50.84) | 22 (50.00) |  |  | 251 (45.89) | 35 (57.28) |  |
| **Physical examination** |  |  |  |  |  |  |  |
| BMI (kg/m2) | 21.00±3.28 | 21.29±3.22 | 0.574 |  | 19.67±3.64 | 19.15±3.19 | 0.287 |
| SBP (mmHg) | 141.15±20.58 | 146.50±24.58 | 0.109 |  | 143.40±22.66 | 141.44±23.88 | 0.525 |
| DBP (mmHg) | 78.56±11.52 | 82.25±11.54 | **0.044** |  | 78.76±11.52 | 76.33±11.03 | 0.118 |
| **Biochemical parameters** |  |  |  |  |  |  |  |
| Blood Urea Nitrogen (mmol/L) | 6.51±1.88 | 7.50±2.54 | **0.015** |  | 6.43±2.00 | 9.39±3.97 | **<0.001** |
| Plasma creatine (mmol/L) ^a^ | 87.00 (74.00-107.00) | 119.50 (89.50-150.25) | **<0.001** |  | 71.00 (59.00-88.00) | 104.00 (82.00-141.50) | **<0.001** |
| Plasma glucose (mmol/L) | 5.47±1.98 | 5.84±2.14 | 0.241 |  | 5.33±1.83 | 5.74±1.59 | 0.091 |
| Total cholesterol (mmol/L) | 3.15±1.20 | 3.02±1.50 | 0.531 |  | 3.74±1.22 | 3.89±1.47 | 0.367 |
| HDL cholesterol (mmol/L) | 1.10±0.31 | 1.08±0.33 | 0.642 |  | 1.19±0.31 | 1.29±0.38 | **0.023** |
| LDL cholesterol (mmol/L) | 1.84±0.68 | 2.20±1.14 | **0.002** |  | 2.10±0.76 | 2.44±0.90 | **0.001** |
| Triglyceride (mmol/L) ^a^ | 1.00 (0.78-1.51) | 1.63 (0.89-3.29) | **<0.001** |  | 1.10 (0.88-1.60) | 1.27 (0.90-2.03) | 0.096 |
| **Disease History** |  |  |  |  |  |  |  |
| Hypertension | 51 (12.3) | 4 (9.1) | 0.535 |  | 90 (16.5) | 16 (26.2) | 0.056 |
| Diabetes mellitus | 8 (1.9) | 0 (0) | 0.746 |  | 7 (1.3) | 1 (1.6) | 0.573 |
| Heart diseases | 21 (5.1) | 2 (4.5) | 0.882 |  | 36 (6.6) | 4 (6.6) | 0.627 |
| Stroke or CVD | 17 (4.1) | 4 (9.1) | 0.259 |  | 11 (2.0) | 4 (6.6) | 0.083 |
| Cancer ^b^ | 1 (0.2) | 0 (0) | 0.904 |  | 1 (0.2) | 0 (0) | 0.900 |
| Chronic nephritis ^b^ | 1 (0.2) | 0 (0) | 0.904 |  | 1 (0.2) | 0 (0) | 0.900 |

**Abbreviations:** SBP, systolic blood pressure; DBP, diastolic blood pressure; BMI, body mass index; HDL, high density lipoprotein; LDL, low density lipoprotein; CVD, cerebrovascular disease. Data are presented as mean ± SD or mean (IQR) for continuous variables and n (%) for categorical variables. Plasma creatine and triglyceride are presented as median and interquartile because of high skew. a: Mann-Whitney U test; b: Fisher’s exact probability test.

# Supplementary Table 4 Association of hyperuricemia with 10-year all-cause mortality by gender among older adults in China (sensitivity analysis)

| **SUA level** | **Individuals** | **Events, n (%)** | **Model 1** | |  | **Model 2** | |  | **Model 3** | |  | **Model 4** | |
| --- | --- | --- | --- | --- | --- | --- | --- | --- | --- | --- | --- | --- | --- |
|  |  |  | **HR (95% CI)** | **p value** |  | **HR (95% CI)** | **p value** |  | **HR (95% CI)** | **p value** |  | **HR (95% CI)** | **p value** |
| **Male** |  |  |  |  |  |  |  |  |  |  |  |  |  |
| ≤ 420μmol/L | 415 | 193 (46.51) | Reference |  |  | Reference |  |  | Reference |  |  | Reference |  |
| > 420μmol/L | 44 | 23 (52.27) | 1.32(0.86-2.03) | 0.209 |  | 1.27(0.80-1.99) | 0.310 |  | 1.15(0.72-1.83) | 0.555 |  | 1.02(0.62-1.66) | 0.949 |
| **Female** |  |  |  |  |  |  |  |  |  |  |  |  |  |
| ≤ 360μmol/L | 547 | 318 (58.14) | Reference |  |  | Reference |  |  | Reference |  |  | Reference |  |
| > 360μmol/L | 61 | 44 (72.13) | 1.86(1.35-2.55) | <0.001 |  | 1.76(1.27-2.45) | 0.001 |  | 1.80(1.29-2.51) | 0.001 |  | 1.82(1.23-2.69) | 0.003 |

**Abbreviations:** BMI, body mass index; CVD, cerebrovascular disease; DBP, diastolic blood pressure; HDL, high density lipoprotein; LDL, low density lipoprotein; SBP, systolic blood pressure. Model 1: crude model; Model 2: further adjusted for age, category of residence, economic income, smoke, drink, physical activity and dietary, in addition to the variables included in Model 1; Model 3: further adjusted for BMI, SBP, DBP, self-reported hypertension, self-reported diabetes, self-reported heart disease, self-reported stroke or CVD and self-reported chronic nephritis, in addition to the variables included in Model 2; Model 4: further adjusted for blood urea nitrogen, plasma creatine, plasma glucose, total cholesterol, HDL cholesterol, LDL cholesterol and triglyceride, in addition to the variables included in Model 3.

# Supplementary Figure 2 Kaplan-Meier survival curves for all-cause mortality categorized by clinical hyperuricemia diagnostic criteria in men (A) and women (B) (sensitivity analysis)





# Supplementary Figure 3. Time-dependent ROC analysis and areas under the curve of the multivariate Cox regression to predict all-cause mortality (sensitivity analysis)


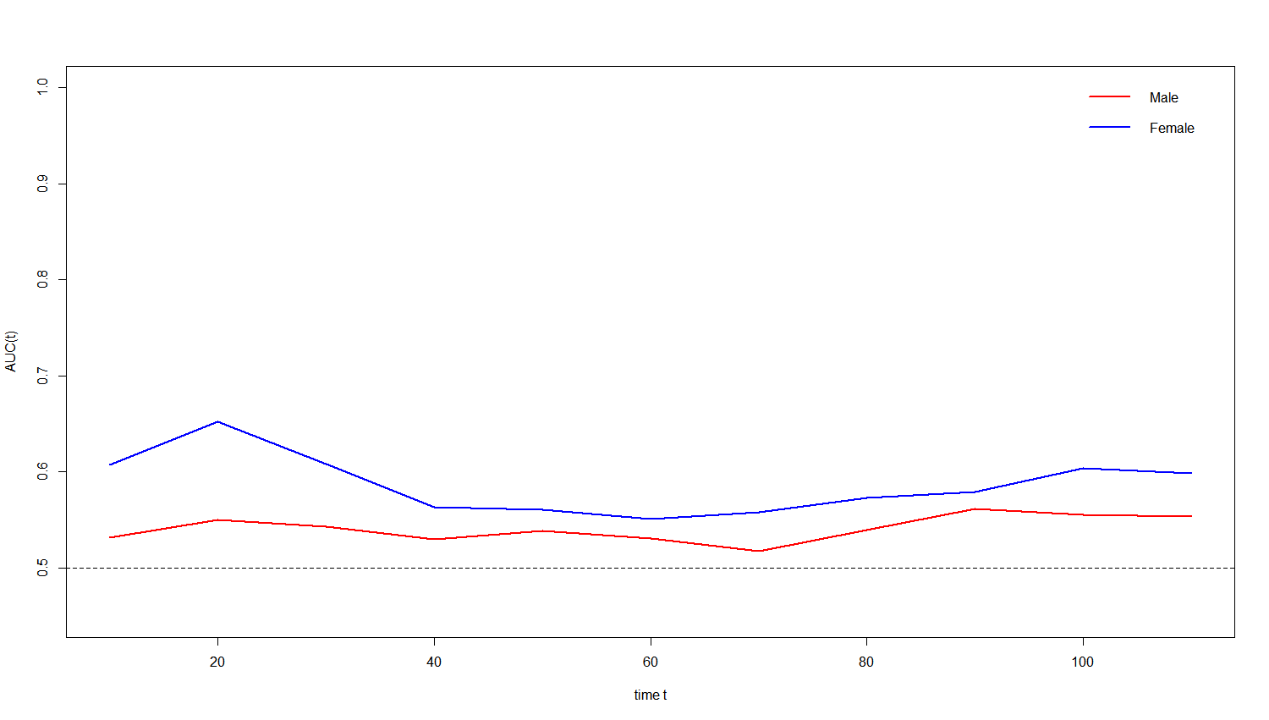


# Supplementary Table 5. Hazard ratios for all-cause mortality among subjects aged≧80 years old in multivariate Cox regression analysis (N=704) (sensitivity analysis)

| Quartiles | Individuals | Events (%) | Person-years | Model 1 | |  | Model 2 | |  | Model 3 | |  | Model 4 | |
| --- | --- | --- | --- | --- | --- | --- | --- | --- | --- | --- | --- | --- | --- | --- |
|  |  |  |  | HR (95%CI) | P value |  | HR (95%CI) | P value |  | HR (95%CI) | P value |  | HR (95%CI) | P value |
| Quartile 1 | 148 | 112 (75.68) | 1545.58 | 1.26 (0.97-1.63) | 0.084 |  | 1.34 (1.03-1.74) | **0.029** |  | 1.35 (1.04-1.76) | **0.026** |  | 1.34 (1.01-1.78) | **0.041** |
| Quartile 2 | 171 | 127 (74.27) | 1440.33 | 1.27 (0.98-1.63) | 0.067 |  | 1.18 (0.91-1.52) | 0.208 |  | 1.20 (0.93-1.56) | 0.158 |  | 1.23 (0.94-1.60) | 0.127 |
| Quartile 3 | 185 | 117 (63.24) | 1415 | Reference |  |  | Reference |  |  | Reference |  |  | Reference |  |
| Quartile 4 | 200 | 146 (73.00) | 1198.50 | 1.43 (1.12-1.83) | **0.004** |  | 1.38 (1.07-1.77) | **0.012** |  | 1.39 (1.08-1.78) | **0.010** |  | 1.31 (1.02-1.69) | **0.041** |

**Abbreviations:** HDL, high density lipoprotein; LDL, low density lipoprotein; SBP, systolic blood pressure; DBP, diastolic blood pressure; BMI, body mass index; CVD, cerebrovascular disease. Model 1: crude model; Model 2: adjusted for age, sex, category of residence, economic income, smoke, drink, physical activity and dietary based on model 1; Model 3: further adjusted for SBP, DBP and BMI, self-reported hypertension, self-reported diabetes, self-reported heart disease, self-reported stroke or CVD and self-reported chronic nephritis based on model 2; Model 4: further adjusted for blood urea nitrogen (BUN), plasma creatine, plasma glucose, total cholesterol (TC), HDL cholesterol, LDL cholesterol and triglyceride (TG) based on model 3.
